# The impact of aquaculture system on the microbiome and gut metabolome of juvenile Chinese softshell turtle (*Pelodiscus sinensis*)

**DOI:** 10.1002/imt2.17

**Published:** 2022-04-05

**Authors:** Xia Ding, Feng Jin, Jiawang Xu, Shulei Zhang, Dongxu Chen, Beijuan Hu, Yijiang Hong

**Affiliations:** ^1^ School of Life Sciences Nanchang University Nanchang Jiangxi China

**Keywords:** aquaculture systems, aquatic animal, host‐associated microbiome, host–microbiome interactions, microbiome–metabolome association

## Abstract

The commercial aquatic animal microbiome may markedly affect the successful host's farming in various aquaculture systems. However, very little was known about it. Here, two different aquaculture systems, the rice–fish culture (RFC) and intensive pond culture (IPC) systems, were compared to deconstruct the skin, oral, and gut microbiome, as well as the gut metabolome of juvenile Chinese softshell turtle (*Pelodiscus sinensis*). Higher alpha‐diversity and functional redundancy of *P. sinensis* microbial community were found in the RFC than those of the IPC. The aquaculture systems have the strongest influence on the gut microbiome, followed by the skin microbiome, and finally the oral microbiome. Source‐tracking analysis showed that the RFC's microbial community originated from more unknown sources than that of the IPC across all body regions. Strikingly, the RFC's oral and skin microbiome exhibited a significantly higher proportion of generalists and broader habitat niche breadth than those of the IPC, but not the gut. Null model analysis revealed that the RFC's oral and skin microbial community assembly was governed by a significantly greater proportion of deterministic processes than that of the IPC, but not the gut. We further identified the key gene and microbial contribution to five significantly changed gut metabolites, 2‐oxoglutarate, *N*‐acetyl‐d‐mannosamine, *cis*‐4‐hydroxy‐d‐proline, nicotinamide, and l‐alanine, which were significantly correlated with important categories of microbe‐mediated processes, including the amino acid metabolism, GABAergic synapse, ABC transporters, biosynthesis of unsaturated fatty acids, as well as citrate cycle. Moreover, different aquaculture systems have a significant impact on the hepatic lipid metabolism and body shape of *P. sinensis*. Our results provide new insight into the influence of aquaculture systems on the microbial community structure feature and assembly mechanism in an aquatic animal, also highlighting the key microbiome and gene contributions to the metabolite variation in the gut microbiome‐metabolome association.

## Highlights

- Different aquaculture system induces distinct phenotype, microbiome assembly, and gut metabolome.
- In brief, different aquaculture system induces distinct tissue‐specific microbiome assembly that alters host fitness in part through gut metabolites.
- Distinct microbial diversity and community structure are driven through different sources and community assembly processes.
- To identify several key genes and microbial species that contribute to five significantly changed gut metabolites.

## Graphical abstract

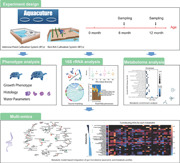

## INTRODUCTION

**Figure 1:**
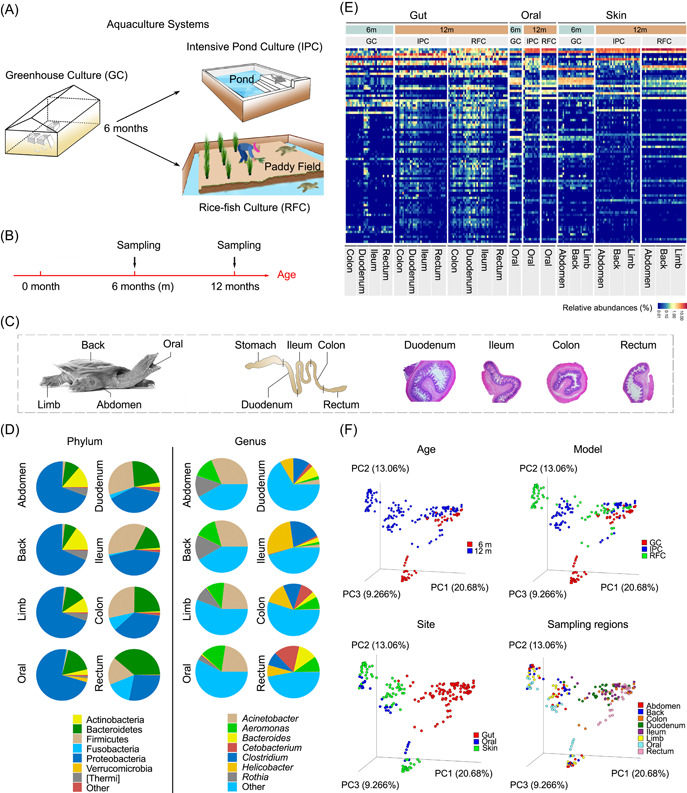
Microbial community compositions in the skin, oral, and gut of juvenile Chinese softshell turtle (*Pelodiscus sinensis*). (A) Schematic illustration of two different aquaculture systems. (B) Sampling time scheme. (C) The spatial structure of the skin, oral, and gut tract, and the sampling regions. (D) Taxonomic analysis showed the phylum and genus classification in each body region. A pie chart of phyla and genus abundances in each body region. (E) Relative abundances of the top 80 genus species in each body region from different culture models and ages. (F) Principal coordinates analysis (PCoA) based on Bray–Curtis distances. Samples are colored by age, culture model, body region, and sampling region. GC, greenhouse culture system; IPC, intensive pond culture; RFC, rice–fish culture

Fish and other aquatic foods (blue foods) provide opportunities for more sustainable diets, but their contribution to food security has been neglected [1]. Small‐scale fishers and aquaculture (SSFA) provide global food for ∼1 billion people [2]. However, aquaculture is now facing unprecedented challenges of climate change, environmental impacts, and human population growth [3, 4]. Farming practices may reshape the watershed structure and the health of aquatic animals. For instance, the intensive pond culture (IPC; high‐intensity rearing) has drastically reduced the biodiversity humans rely on and causes environmental problems and infectious diseases. Therefore, there is an urgent need to develop low input and low impact aquaculture [5]. Integrated agriculture–aquaculture (IAA) is a way to simultaneously produce grain and animal protein in the same place, placing a high value on the ecological environment and promoting sustainability [6]. Rice–fish culture (RFC) is one of the most popular IAA systems across the world, especially in tropical and subtropical Asia, which captures and cultures aquatic organisms from paddy fields (Figure 1A). Asia has a tradition and a long history of 1700 years in RFC practice [7]. Regarding the general scale of RFC, China is the primary producer. RFC is now one of the most essential agricultural systems in China. Several aquatic species, including Chinese softshell turtles, carps, shrimp, and frogs, are being farmed in rice fields [8].

The aquatic animal body is home to trillions of microbial cells with remarkably variable and dynamic compositions and functions. Numerous pieces of evidence have revealed the importance of the interactions between animal and their associated microbiota. Different body regions, such as the gut, oral, and skin, have distinct microbial inhabitants. The host‐associated microbiome provides essential functions for the physiology and fitness of its host [9]. The gut microbiome helps host nutrition absorption, training immunity, and intestinal development [10]. Similar to the microbiome in the gut, skin microbes have essential roles in protecting hosts from pathogen invasion and training the host immune system [11]. The oral microbiome plays a crucial role in periodontitis, caries, and systemic diseases [12]. Microbes are greatly essential to aquaculture and can be added artificially to play different roles. On the other hand, controlling the aquaculture microbiota has always been important in the high‐intensity rearing model. Therefore, understanding and manipulating the microbial community in aquaculture environments has great potential. However, our current lack of knowledge of aquaculture microbiota and the overall ecology of these systems hinders the successful management of the microbiota in aquaculture environments.

The composition of the host‐associated aquaculture microbiome exhibits a great deal of plasticity in response to environmental change. These changes may be very subtle, may only activate or inactivate certain metabolic pathways, and may lead to changes in the composition and function of the entire microbial community. Much of this diversity and plasticity of the microbiome remains unexplained, although diet, environment, host genetics, and early microbial exposure have been intensively studied [13]. Even in the face of the same environmental disturbance, the microbiome response in different niches varies. Given the importance and complexity of the host‐associated microbiome, it is vital to comprehend factors and ecoprocesses driving the compositional patterns and functionality of the aquaculture microbiome within various body habitats. Farming practices strongly reshape the composition of the host‐associated microbiome [14, 15]. To better manage aquatic animals' health and disease, there is a need to mechanically understand how the aquaculture systems affect commercial aquatic animals' microbiome and host fitness.

The Chinese softshell turtle, *Pelodiscus sinensis*, is one of the most commercially essential aquatic species with high nutritional and pharmaceutical values in Asia, especially in China, Japan, and Korea [16, 17]. It is generally considered that the RFC favors biodiversity maintenance, and manufactures food of high nutritional quality, compared with the IPC system. With high market prices and nutritional quality resulting from a relatively limited RFC system, *P. sinensis* from the RFC was considered to be a high‐value aquatic species in Asia. Therefore, It is necessary to systematically evaluate the aquaculture system's effects on *P. sinensis* and the environment. Furthermore, *P. sinensis* locates between ectothermic amniotic animals (fishes and amphibians) and endothermic amniotic animals (birds and mammals), which play an essential role in vertebrate evolution [18, 19]. *P. sinensis* is a good model animal in genetic evolution, phylogeny, and biodiversity. Therefore, *P. sinensis* is a vital model animal in the research works of RFC and IPC systems. However, it is rare to make explicit comparisons of the gut, oral, and skin microbiomes from the same individuals of aquatic animals, and it is largely unknown whether these host‐associated microbiomes will change on the same spatial scale in response to similar environmental cues.

The primary aim of this study was to (1) comprehensively analyze the microbiome structure feature of juvenile Chinese softshell turtle, *P. sinensis*, (2) make a direct comparison between the response of the skin, oral, and gut microbiomes to changes in two different aquaculture environments (the RFC and IPC), (3) evaluate how the various aquaculture systems affect the profiles of the gut microbiome and metabolome, and interaction between microbe, metabolic, and host. To resolve these issues, the effect of two various aquaculture systems on the microbiome of *P. sinensis* was systematically explored. The skin (back, limb, and abdomen), oral, and gut (duodenum, ileum, colon, and rectum) microbiome as well as gut metabolome analysis were conducted in *P. sinensis* from the RFC and IPC, respectively. This will provide more insights into the structure feature and functional roles of the microbiome of commercial aquatic species in regulating host fitness response to environmental change and will enhance further aquaculture management.

## RESULTS

### Microbiome structure of juvenile Chinese softshell turtle (*P. sinensis*)

In May, 6‐month‐old *P. sinensis* was transferred from the greenhouse culture (GC) to the pond and paddy fields for the IPC and RFC, respectively (Figure 1A and Table S1). According to the gut anatomical and histological structure of *P. sinensis*, the guts comprise four parts, including the duodenum, ileum, colon, and rectum (Figure S1). The skin (back, limb, and abdomen), oral, and gut (duodenum, ileum, colon, rectum, and whole gut) microbiome analysis was conducted using 16S rRNA sequencing of the 6‐ and 12‐month‐old *P. sinensis* (Figure 1B,C and Figure S1). A total of 23,184 features and 10,983,297 reads were obtained among 240 samples with a mean reads 45,763 (range: 22,000–55,039). Across body regions, the composition of the microbial communities varied significantly (Kruskal–Wallis test, *p* < 0.05; Figure 1D). Amplicon sequence variants (ASVs) of 2500, 2481, 2681, 2192 ASVs were identified within the oral, abdomen, limb, and back, and 837 ASVs were shared among four regions. ASVs of 9102, 5708, 4851, and 2561 were identified within the duodenum, ileum, colon, and rectum, and 761 ASVs were shared among all four regions. It was noted that the shared ASVs accounted for 14.4% and 4.8% relative abundance in skin/oral and gut regions, respectively (Figure S2).

At the phylum level, the skin (back, limb, and abdomen) and oral were dominated by *Proteobacteria, Bacteroidetes, Actinobacteria*, and *Thermi*, and the gut (duodenum, ileum, colon, and rectum) was dominated by *Proteobacteria, Bacteroidetes, Firmicutes*, and *Fusobacteria* (Figure 1D and Table S2). At the genus level, the skin (back, limb, and abdomen) and oral were dominated by *Acinetobacter, Aeromonas*, and *Rothia*, and the gut (duodenum, ileum, colon, and rectum) was dominated by *Clostridium, Helicobacter, Cetobacterium, Bacteroides*, and *Aeromonas* (Figure 1D and Table S3). There were notable differences in the proportions of various genus across the body regions, age, and aquaculture system (Kruskal–Wallis test, *p* < 0.05; Figure 1E and Table S4). However, there is no such significant difference in the microbiome in different parts of the skin or gut (Kruskal–Wallis test, *p* > 0.05). In the oral and skin, the microbiome of the RFC and IPC has a significantly greater proportion of *Acinetobacter* than that of the GC (Kruskal–Wallis test, *p* < 0.05). The microbiome of the GC and IPC had a significantly greater proportion of *Aeromonas* than that of the RFC (Kruskal–Wallis, *p* < 0.01). In the gut, the microbiome of the IPC has the greatest proportion of *Helicobacter*, and the microbiome of GC has the greatest proportion of *Clostridium* (Kruskal–Wallis test, *p* < 0.01).

To investigate the patterns of separation between microbial compositions, principal coordinates analysis (PCoA) based on Bray–Curtis distance was performed. The results showed that the microbiomes within the age, culture model, and region were separated (Adonis, *p* < 0.001, Figure 1F). Permutational multivariate analysis of variance (PERMANOVA) showed that the body region comprises the largest variation source within the microbiome data (24.6%, *p* < 0.001), the culture model comprises the second variation source (16.2%, *p* < 0.001), and age comprises the third variation source (9.8%, *p* < 0.001; Table S5). Next, how the culture model influenced the site‐specific microbial communities were analyzed.

### Variation in microbiome alpha‐ and beta‐diversity in RFC and IPC

**Figure 2:**
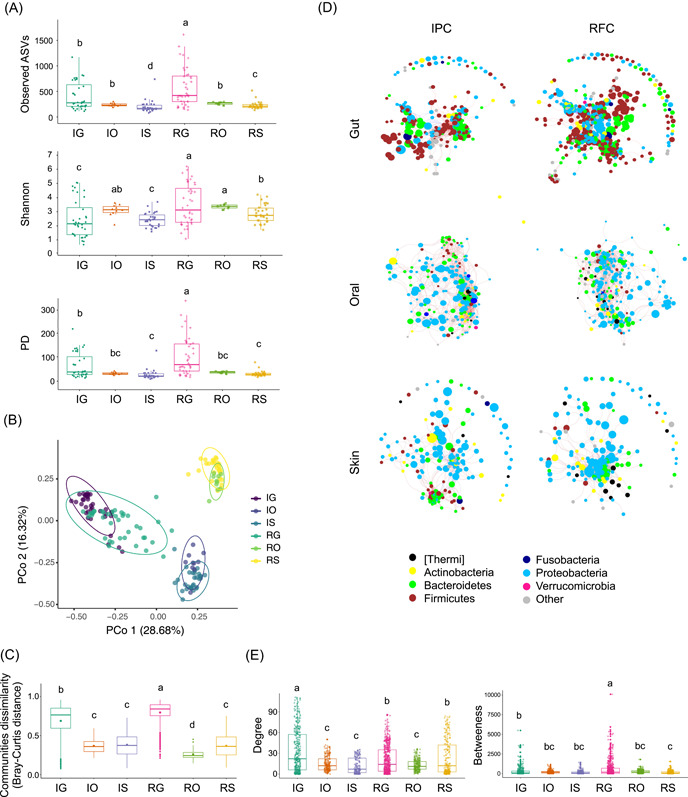
Microbial diversity and community co‐occurrence networks in the RFC and IPC. (A) Alpha‐diversity indices (number of observed ASVs, Shannon, and phylogenetic diversity [PD]) in the skin, oral, and gut from the RFC and IPC. (B) Differences in *Pelodiscus sinensis* skin, oral, and gut microbiome beta‐diversity associated with the RFC and IPC. PCoAs based on Bray–Curtis dissimilarity distance. (C) Microbial community dissimilarity in the skin, oral, and gut from the RFC and IPC. Each box plot represented the skin, oral, and gut within the RFC and IPC. Significant differences were compared between each box. (D) Microbial community co‐occurrence networks with strong Spearman's correlation values (*p* < 0.5 and abs (*r*) > 0.6). Network nodes are colored by phylum and size based on the relative abundance of the ASVs (log 10 transformed relative abundance). The negative correlations of edges are colored blue, and the positive correlations of edges are colored red. (E) Node‐edge statistic of microbial community co‐occurrence networks. Significant differences (Kruskal–Wallis test, *p* < 0.05) are shown by different letters. ASV, amplicon sequence variant; IG, gut in the IPC; IO, oral region in the IPC; IPC, intensive pond culture; IS, skin in the IPC; PCoA, principal coordinates analysis; RFC, rice–fish culture; RG, gut in the RFC; RO, oral region in the RFC; RS, skin in the RFC

To compare the response of the skin, oral, and gut microbiomes to the changes in two separate aquaculture environments, the variation in microbiome alpha‐ and beta‐diversity was analyzed. The alpha diversity of the RFC was significantly higher than that of the IPC in gut and skin regions (Kruskal‐Wallis test, *p* < 0.05), but the alpha diversity between the RFC and IPC was not varied significantly in the oral region (Kruskal–Wallis test, *p* > 0.05; Figure 2A). In more detail, the gut microbial Shannon index, the number of observed ASVs, and phylogenetic diversity in the RFC were higher than those of the IPC. The skin microbial Shannon index and the number of observed ASVs in the RFC were higher than those of IPC. Moreover, alpha‐diversity of the gut microbiome was significantly higher than that of oral and skin microbiome in both RFC and IPC (Kruskal–Wallis test, *p* < 0.05; Figure 2A).

The variation in the microbiome community structure reinforces the pattern of alpha‐diversity. First, the IPC microbiome samples from the gut, oral, and skin were separated from the RFC principally on the second PCoA axis (PERMANOVA, *p* < 0.05; Figure 2B). The permutation‐based statistical test showed that all the microbiome groups were significantly different from other groups (pairwise PERMANOVA, *p* < 0.05; Table S6 and Figure 2B). Next, the Bray–Curtis and Jaccard dissimilarity distance showed that the gut microbiome communities of the RFC exhibited a greater dissimilarity than that of the IPC (Figure 2C). Discrepancies in the gut microbiome communities were greater than those in oral and skin microbiome communities in both culture models.

### Microbial co‐occurrence networks in RFC and IPC

A microbial ASV co‐occurrence network was generated using significant correlations to explore the more detailed changes in the potential interactions among microbiota between the RFC and IPC. The gut has more edges and vertices than the skin and oral microbial networks (Table S7). For the gut and skin regions, the network degree between the RFC and IPC was significantly different, but not oral (Kruskal–Wallis test, *p* < 0.05; Figure 2D,E). The network between RFC and IPC was significantly different in the gut, but not oral and skin (Kruskal–Wallis test, *p* < 0.05; Figure 2D,E). For the gut and skin regions, the number of negative correlations of RFC was significantly higher than that of the IPC (Wilcoxon test, *p* < 0.05; Figure 2D and Table S7). Additionally, how the network hub (keystone) responded to the cultural model was observed. For the skin regions, the network hubs were dominated by *Bacteroidetes* and *Firmicutes* in the IPC, whereas *Proteobacteria* dominated the network hubs in the RFC (Figure S3). Altogether, the results of diversity and networks indicated that the gut microbiome was the greatest difference between RFC and IPC, followed by the skin microbiome, and finally the oral microbiome.

### Taxonomic and functional differences of the gut, oral, and skin microbiomes between RFC and IPC

**Figure 3:**
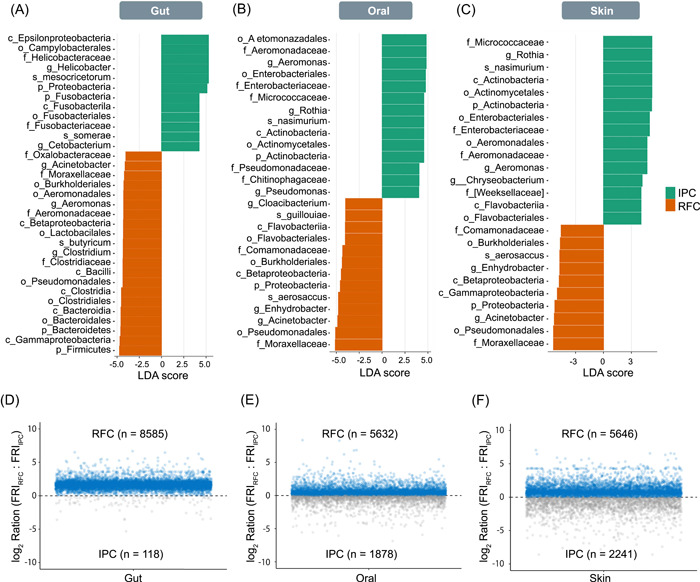
Taxonomic and functional differences of skin, oral, and gut microbiome between the RFC and IPC. (A–C) Linear discriminant analysis (LDA) effect size (LEfSe) analysis revealed significant microbial differences in the (A) skin, (B) oral, and (C) gut between RFC and IPC. LDA scores >4 and *p* < 0.05 are shown. (D–F) Comparing the functional gene redundancies of gut (D), oral (E), and skin (F) microbiome communities between the RFC and IPC. A high FRI indicates that a specific function is almost ubiquitous in all community members. In contrast, a low FRI suggests that the function is present in a few closely related species. A log ratio greater than 0 indicates that a function is more redundant in the RFC, a log ratio smaller than 0 indicates that a function is more redundant in the IPC. All predictions were made using a 97% similarity cut‐off. FRI, functional redundancy index; IPC, intensive pond culture; RFC, rice–fish culture

Changes in the gut, oral, and skin microbiome between RFC and IPC using linear discriminant analysis (LDA) effect size (LEfSe) analysis were further compared based on ASVs. The histogram of the LDA scores revealed a clear differential abundance between the RFC and IPC. For the gut, the LDA scores showed that the relative abundance of phylum *Firmicutes, Bacteroidetes, Actinobacteria*, class *Betaproteobacteria*, order *Lactobacillales, Burkholderiales*, and *Pseudomonadales*, family *Lachnospiraceae* and *Moraxellaceae*, genus *Lactobacillus, Clostridium, Acinetobacter, Enterococcus, Rothia, Sphingomonas, Ruminococcus, Roseburia, Prevotella, Bifidobacterium, Eubacterium, Faecalibacterium, Bacteroides*, and *Aeromonas* were much enriched in the RFC (LEfSe, *p* < 0.05), the relative abundances of phylum *Proteobacteria, Fusobacteria*, genus *Helicobacter, Cetobacterium, Methylobacterium*, were much enriched in the IPC (LEfSe, *p* < 0.05; Figures 3A and S4). For the oral, the relative abundance of phylum *Proteobacteria*, order *Burkholderiales* and *Pseudomonadales*, family *Moraxellaceae*, genus *Acinetobacter* were enriched in the RFC (LEfSe, *p* < 0.05), the relative abundance of order *Aeromonadales* and *Enterobacteriales*, genus *Aeromonas* was much enriched in IPC (LEfSe, *p* < 0.05; Figure 3B). For the skin, the relative abundance of order *Burkholderiales* and *Pseudomonadales*, family *Moraxellaceae*, genus *Acinetobacter* were enriched in the RFC (LEfSe, *p* < 0.05), the relative abundance of family *Micrococcaceae* and genus *Rothia* and *Aeromonas* were much enriched in the IPC (LEfSe, *p* < 0.05; Figure 3C). It was observed that the order *Burkholderiales* and *Pseudomonadales*, family *Moraxellaceae*, genus *Acinetobacter* were common enriched species in RFC among gut, oral, and skin regions.

To understand the difference in microbial community functionally redundancy, the functional redundancy index (FRI) in Tax4Fun2 was calculated to compare functional redundancy in the gut, oral, and skin microbial communities between the RFC and IPC. For the gut, oral, and skin, the RFC's FRI was pronounced higher than that of the IPC (Figure 3D–F). Particularly, the FRI had very pronounced differences in the gut microbial communities between the RFC and IPC. The results indicated that the RFC's function is more redundant than that of the IPC in all gut, oral, and skin microbial communities, especially the gut microbial community.

### Source tracking analysis of the microbiome in RFC and IPC

**Figure 4:**
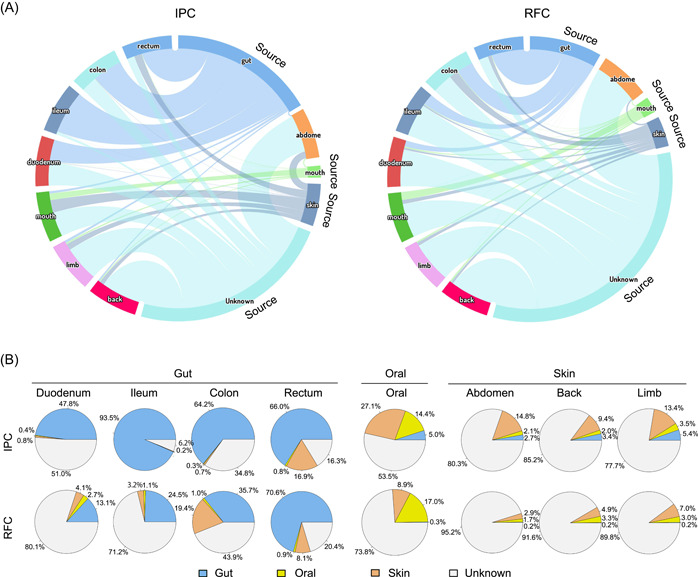
Bayesian source‐tracking for the microbiome communities in the RFC and IPC. (A) Estimated source contributions from each region are shown for each sample. (B) Source‐tracker proportion estimates. Six‐month‐old *Pelodiscus sinensis* skin, oral, and gut as sources to predict putative microbiome sources of 12‐month‐old *P. sinensis* skin (back, limb, and abdomen), oral, and gut (duodenum, ileum, colon, rectum, and whole gut) in RFC and IPC. IPC, intensive pond culture; RFC, rice–fish culture

To analyze the reason why microbial community in the RFC displayed significantly higher alpha‐diversity and functionally redundancy compared with those of the IPC, dynamic Bayesian inference neural information flow networks was used. Six‐month‐old *P. sinensis* skin, oral, and gut were used as sources to predict putative microbiome sources of 12‐month‐old *P. sinensis* skin (back, limb, and abdomen), oral, and gut (duodenum, ileum, colon, rectum, and whole gut) in RFC and IPC. Results revealed that the microbial community originated from several environments, including the skin, oral, gut, and unknown sources, and unknown sources were the main source of the microbial community for both the RFC and IPC (Figure 4A and Table S8). Unknown sources were more likely to be the surrounding water, soil, and environments. The gut microbiome in the RFC and IPC mainly originated from the gut and unknown sources, partly originating from the skin and mouth (Figure 4B and Table S8). The skin and oral microbiome in the RFC and IPC mainly originated from the skin, oral, and unknown sources, but a small part of the skin and oral microbiome in the pond culture model also originated from gut sources. The proportion of unknown sources was the highest on the skin microbiome, followed by the oral microbiome, and finally the gut microbiome. Unknown sources were identified as the biggest contributor to microbiome sources, particularly in the skin and oral samples. Mainly, the microbial communities in the RFC originated from more unknown sources than that of the IPC among all body regions (Figure 4B and Table S8).

### Microbial community assembly process in the RFC and IPC

**Figure 5:**
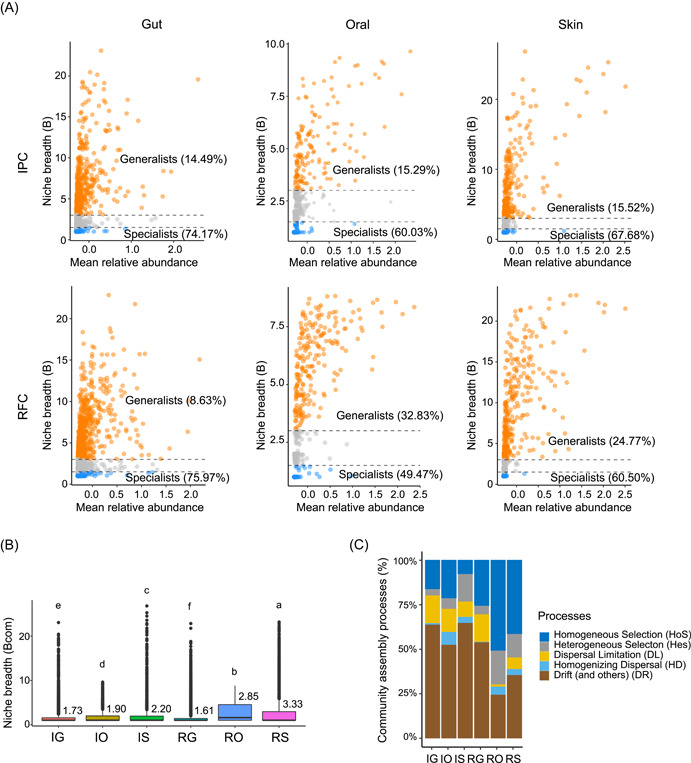
The microbial community assembly processes. (A) The mean abundances of ASVs of microbiome versus habitat niche breadth. The ASVs of temporal generalists are colored orange, the ASVs of specialists are colored blue. (B) Boxplots illustrating the mean habitat niche breadth (Bcom). Significant differences (Kruskal–Wallis test, *p* < 0.05) are indicated by different letters. (C) The percentage of turnover in the skin, oral, and gut microbial communities in RFC and IPC was governed by deterministic processes (homogeneous and heterogeneous selection) and stochastic processes (dispersal limitation, homogenizing dispersal, and drift). ASV, amplicon sequence variant; IG, gut in the IPC; IO, oral region in the IPC; IPC, intensive pond culture system; IS, skin in the IPC; RFC, rice–fish culture systems; RG, gut in the RFC; RO, oral region in the RFC; RS, skin in the RFC

The microbial community was partitioned into temporal generalists and specialists. It was found that oral and skin temporal generalists were much higher than gut temporal generalists in RFC and IPC (Figure 5A). There were comparable proportions of gut temporal generalists (10.49% vs. 8.63%) and specialists (74.17% vs. 75.97%) between RFC and IPC. However, the RFC's oral and skin temporal generalists occupied 32.83% and 24.77% of the total diversity, which was much higher than those of the IPC, where they merely accounted for 15.29% and 15.52%. Consistently, the mean community niche breadth (Bcom) for oral and skin microbial communities was significantly wider than that of gut microbial communities in both the RFC and IPC (Kruskal–Wallis test, *p* < 0.05; Figure 5B). The mean Bcom for oral and skin microbial communities of RFC was significantly wider than that of the IPC (Kruskal–Wallis test, *p* < 0.05), but the mean Bcom for gut microbial communities of the RFC was light narrower than that of the IPC (Kruskal–Wallis test, *p* < 0.05). The results indicated that the oral and skin microbiome was more metabolically flexible at the community level than the gut microbiome. In addition, the oral and skin microbiome of the RFC was more metabolically flexible at the community level than that of the IPC.

The relative roles of deterministic processes (homogeneous and heterogeneous selection) and stochastic processes (dispersal limitation, homogenizing dispersal, and drift) in governing the skin, oral, and gut microbial communities in the RFC and IPC were quantified. The community assembly process was divided into two patterns according to the different niches. For skin and oral, drift was the most important process in governing oral (52.3%) and skin (64.6%) microbial communities in the IPC. In comparison, the homogeneous selection was the most important process in governing oral (51.0%) and skin (41.6%) microbial communities in the RFC. For the gut, drift was the most critical process in governing gut microbial communities in both IPC and RFC, with an average relative importance of 63.5% and 53.6%. Additionally, it was observed that the oral and skin microbial communities of the RFC were governed by a greater proportion of deterministic processes than that of the IPC. Still, the gut microbial communities of the RFC were governed only by a weak greater proportion of deterministic processes than that of the IPC.

### Key microbiome and gene contributions to metabolite variation in gut microbiome–metabolome association

**Figure 6:**
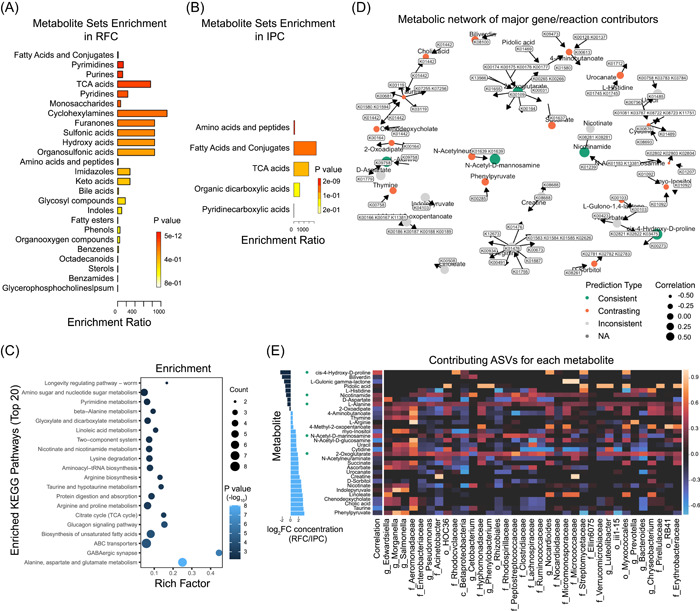
Metabolic model‐based integration of gut microbiome taxonomic and metabolic profiles. (A,B) Metabolite set enrichment analysis (MSEA) was performed using the significant enrichment metabolite sets in the RFC (A) and IPC (B). The enrichment ratio is shown on the *x*‐axis. The horizontal bar's colors indicate pathway enrichment significance (Fisher's exact test, *p* < 0.001). (C) Metabolic enrichment analysis based on KEGG full library performed using the significant changes in gut metabolites between the RFC and IPC. The enrichment factor is shown on the *x*‐axis. Circle colors indicate pathway enrichment significance. Circle size indicates the extent of pathway impact. (D) The metabolic network of major genes/reactions was found to be the key contributor to each metabolite. The color and size of each node indicate how well that metabolite was predicted by community metabolic potential. The well‐predicted metabolites are defined as those for which the variation in CMP scores is significantly correlated (using a Mantel test) with variation in measured metabolite abundance at an FDR < 0.01. Antipredicted metabolites are similarly defined as those for which variation in CMP scores is significantly negatively correlated with variation in measured metabolite abundances (FDR < 0.01). (E) MIMOSA‐model prediction of microbe involved in production or degradation of specific metabolites. The heatmap visualization of the potential taxonomic contributors for all metabolites. The columns correspond to microbiota in the pond and rice–fish culture system. Rows correspond to the metabolites detected using LC‐MS/MS, which were significantly consistent with metabolic potential at FDR < 0.05. The segments along the left side show the fold change of metabolite concentration in RFC and IPC samples. The segments in the median show the correlation magnitude. The metabolites marked with green circles indicate predicted consistent type. CMP, community metabolic potential; FDR, false discovery rate; IPC, intensive pond culture; LC‐MS, liquid chromatography–mass spectrometry; MIMOSA, Model‐based Integration of Metabolite Observations and Species Abundance; MS, mass spectrometry; RFC, rice–fish culture

The metabolite set enrichment analysis (MSEA) and metabolic pathway analysis (MetPA) were conducted on all 82 identified significantly changed metabolites in the gut (variable importance in projection (VIP) > 1, unpaired Student's *t* test, *p* < 0.05; Figure S5), proposing large‐scale pathway changes of metabolism in the gut between the RFC and IPC [20, 21]. There were higher concentrations of fatty acids and conjugates, pyrimidines, purines, TCA acids, pyridines, and monosaccharides in the gut of RFC (Fisher's exact test, *p* < 0.001; Figure 6A and Table S10). There were higher concentrations of amino acids and peptides in the gut of the IPC (Fisher's exact test, *p* < 0.001; Figure 6B and Table S10). The top‐altered pathways were involved in the alanine, aspartate, and glutamate metabolism, GABAergic synapse, biosynthesis of unsaturated fatty acids, glucagon signaling pathway, citrate cycle (TCA cycle), arginine, and proline metabolism (Figure 6C and Table S11).

The multi‐omic data were analyzed using the Model‐based Integration of Metabolite Observations and Species Abundances (MIMOSA) to investigate the relationship between microbiome and metabolome from gut contents. Overall, 5 out of 32 variable metabolites were significantly (false discovery rate [FDR] 0.1) consistent with metabolic potential (15.6%), and 16 were significantly contrasting (50%). Well‐predicted (consistent) metabolites included 2‐oxoglutarate, *N*‐acetyl‐d‐mannosamine, *cis*‐4‐hydroxy‐d‐proline, nicotinamide, and l‐alanine. The community metabolic potential (CMP) scores were calculated to identify the potential gene, reaction, and species contributors for each metabolite, most metabolites had several key gene contributors which encoded enzymes catalyzing production and/or degradation of metabolite (Figure 6D and Table S12). Specifically, those well‐predicted metabolites were associated with amino acid metabolism, cofactors and vitamin metabolism, and energy metabolism, which were essential categories of microbe‐mediated processes in the gut (Figure S6). Key species contributor analysis indicated that ASVs in the class *Betaproteobacteria*, family *Peptostreptococcaceae, Ruminococcaceae, Aeromonadaceae*, and *Lachnospiraceae*, and genus *Bacteroides* and *Pseudomonas* contributed to the increased synthesis from 2‐oxoglutarate, ASVs in the family *Peptostreptococcaceae* and *Rhodospirillaceae* contributed the increased synthesis from *N*‐acetyl‐d‐mannosamine (Figure 6E and Table S13,14). Additionally, ASVs in the order Myxococcales, family *Streptomycetaceae* and *Rhodocyclaceae*, the genus *Nocardioides* contributed to the increased degradation of *cis*‐4‐hydroxy‐d‐proline, ASVs in the family *Peptostreptococcaceae* and *Lachnospiraceae*, genus *Cetobacterium* contributed the increased degradation of nicotinamide.

### Growth conditions and phenotypic traits of *P. sinensis* in RFC and IPC

**Figure 7:**
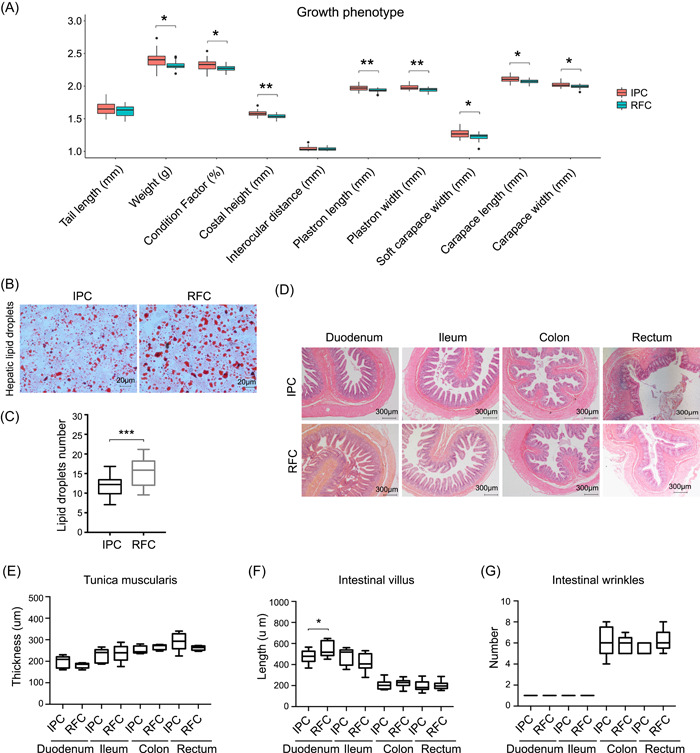
Growth conditions and phenotypic traits of *Pelodiscus sinensis* in the RFC and IPC. (A) The growth‐related phenotypic traits of *P. sinensis* in two agriculture culture systems. Data were log_10_ transformed. (B) Representative oil red O staining of hepatic lipid droplets. Scale bar: 20 μm. (C) The number of hepatic lipid droplets in each treatment condition. (D) Representative H&E staining of gut segments (duodenum, ileum, colon, and rectum). Scale bar: 300 μm. (E) The thickness of tunica muscularis in each treatment condition. (F) The length of the intestinal villus in each treatment condition. (G) The number of intestinal wrinkles in each treatment condition. *p < 0.05, **p < 0.01, and ***p < 0.001 (*t* test). H&E, hematoxylin–eosin; IPC, intensive pond culture; RFC, rice–fish culture

To compare the influence of culturing environment on microbiota–host–environment interaction, the growth traits, and histological features of *P. sinensis*, and the physical parameters of water in the RFC and IPC were detected. There were significant variations in growth‐related phenotypic traits of *P. sinensis* between the two agricultural culture systems (*t* test, *p* < 0.05), the development of *P. sinensis* in RFC was significantly slower than that of IPC (Figure 7A). Further, the gut and liver histological changes were evaluated; RFC significantly promoted the accumulation of hepatic LDs (*t* test, *p* < 0.001; Figure 7B,C). However, there were no significant histological changes in the gut (duodenum, ileum, colon, and rectum) tissues (*t* test, *p* > 0.05), except the length of the intestinal villus in the duodenum tissue in RFC was significantly greater than that of IPC (*t* test, *p* < 0.05; Figure 7D–G). Additionally, it was observed that there were no significant differences in the environmental factors (e.g., the pH, DO, TOC, TON, and NO_3_^−^) and plankton of water between the RFC and IPC (*t* test, *p* > 0.05; Figure S7 and Table S15).

## DISCUSSION

### The microbiome diversity and stability of *P. sinensis* in RFC and IPC

The PCoA analysis illustrated that the microbial communities from the RFC and IPC were significantly separated (Adonis, *p* < 0.001; Figure 1F and Table S5). The alpha‐diversity of the RFC was significantly higher than that of the IPC (Figure 2A). Wu et al. observed a similar result that the gut microbiome from paddy fields has a relatively higher richness than that from the pond [15]. In the IPC, the relatively homogeneous hydrographic condition caused relatively low alpha‐diversity. The complex spatial structure (i.e., paddy field, water channel, rice) in the RFC caused higher alpha‐diversity. This is supported by the results of the source‐tracking analysis. It was observed that the microbial community in the RFC originated from more unknown sources than that of IPC (Figure 4), which indicated that *P. sinensis* in the RFC gained more microbes from the more complex surrounding environment. Therefore, interaction with various surrounding environments could shape the distinct symbiotic microbial community of *P. sinensis*.

The microbial ecosystem stability is considered critical for host health and well‐being but is poorly understood [22, 23]. Microbiome metabolism is highly flexible. Thus, the microbiome plays a front‐line role in determining ecosystem response to environmental change. We aimed to understand the extent to which the aquaculture model affected the host‐associated microbiome stability. It is generally accepted that there is a positive relationship between biodiversity and stability [24, 25]. Our data showed that *P. sinensis* microbial community in the RFC displayed significantly more alpha‐diversity than that of the IPC (Figure 2A), suggesting that the RFC's microbiome was more stable than the IPC's microbiome. However, a study using a mathematical model to investigate the relationships between diversity and stability showed contradictory results, especially diversity comparisons between healthy and pathological states [26]. The measurement of microbial diversity is a starting point rather than an “answer” to community outcomes [27]. To understand the complex ecological community stability and its role in host health, our approach was broadened to analyze our microbiome data using the network interaction and functional redundancy. Coyte et al.'s model predicted that increased ecological competition thought to introduce network negative‐feedback loops would enhance microbial community stability [22]. In gut and skin regions, the number of network negative correlations of the RFC was significantly higher than that of the IPC (Figure 2D and Table S7). Additionally, recent work indicated the importance of functional redundancy‐induced stability of gut microbiota [28]. Our data showed that *P. sinensis* microbial community in the RFC displayed significantly more functional redundancy than that of the IPC, especially the gut microbial community (Figure 3D–F). Overall, it was suggested that RFC may benefit host fitness by promoting host–microbiome stability.

### The microbiome assembly mechanism of *P. sinensis* in RFC and IPC

Microbial community assembly is essential to understand the mechanisms by which microbial communities regulate ecosystem‐level functions [29]. We further investigated the community assembly process driving the identity and abundance of the microbiome in the RFC and IPC. First, the potential sources of the microbiome in the RFC and IPC were analyzed. In both the RFC and IPC, the skin and oral microbiome mainly originated from the skin, oral, and unknown sources, and the gut microbiome mainly originated from the gut and unknown sources (Figure 4 and Table S8). However, the microbial communities in the RFC originated from more unknown sources than that of the IPC across all body regions, especially in the skin and oral. The more unknown sources input was observed in the RFC, indicating that the microbial communities in the RFC may get more heterogeneous species from the surrounding environment (such as aquatic and soil environment, rice, and food), causing the higher alpha‐diversity and functional redundancy (Figures 2A and 3D–F). This is supported by the results of the neutral community model. Higher deterministic assembly processes were observed in the RFC, especially in the skin and oral (Figure 5C), indicating that the microbial communities in the RFC might comprise more heterogeneous species. Deterministic processes may overwhelm stochastic processes in systems with more environmental variation [30]. The pond water flow and relative homogeneous hydrographic conditions in the IPC can cause a higher level of microbial dispersal, while the complex spatial structure and relative heterogeneous spatial conditions in the RFC can cause a higher level of microbial environmental selection. Because of the different microbiome tolerance to changing environments [31, 32], the species were further divided into habitat generalists and specialists. The oral and skin microbial community in the RFC harbored a higher proportion of generalists and fewer specialists than that of the IPC (Figure 5A). Consistently, the skin and oral microbial communities in the RFC exhibited significantly wider niche breadth than that of the IPC (Figure 5B). It is generally believed that habitat generalists with wider niche breadth are more metabolically flexible at the community level and adapt to the wider environment [31, 33]. A potential explanation is that the RFC was a more heterogeneous environment than the IPC, causing more unknown environmental sources and more environment filtering, finally causing a higher alpha‐diversity and metabolically flexible microbial communities to adapt to the varied heterogeneous varied environment. Moreover, the microbial assembly of generalists and specialists is controlled by a different process [34]. Many studies observed that the assembly of generalists and specialists was strongly shaped by the neutral process and environment selection, respectively [35, 36]. Our data indicated that the oral and skin microbiome of the RFC harbored a higher proportion of generalists than that of the IPC. Still, homogeneous selection was the most critical process in governing oral and skin microbial communities in the RFC (Figure 5). Previous conclusions are based on the free‐living microbiome. The controversial finding remains an open question about how assembly processes affect the distribution of generalists and specialists of the host‐associated microbiome in different aquaculture systems. It was suggested that different from the free‐living microbiome, the host‐associated microbial community strengthened the environmental selection process to shape symbiotic microbiome forming a more proportion of generalists under a diverse heterogeneous environment.

Previous studies also reported that the microbiome assembly processes depended on particular environment types, conditions, and microbial traits [37]. Moreover, the oral and skin microbial community harbored a significantly higher proportion of generalists and wider niche breadth than the gut microbial community (Figure 5A). As a result, the skin and oral microbiome were more metabolically flexible, and adaptable to the broader environment, also more resistant to environmental impact than the gut microbiome. It is well known that the skin and oral region are open‐field, but the gut region is an anaerobic close‐field in which the microbiome is strongly driven by electron acceptor availability [38]. The low dissolved oxygen in the gut region causes strong environmental filtering of specialists, resulting in a significantly higher proportion of specialists and a lower proportion of generalists were observed in the gut than oral and skin (Figure 5A). It is believed that generalists were more versatile but less efficient than specialists, while specialists performed more effectively but fewer activities than generalists. The gut is the most active metabolic region of the body, which requires an efficient microbiome for metabolite synthesis and degradation. Therefore, the gut harbored a higher proportion of specialists to perform restricted but efficient metabolism, whereas skin and oral harbored a higher proportion of generalists with broad metabolic capabilities to adapt to wide‐ranging environmental conditions.

### The microbiome composition of Chinese softshell turtle (*P. sinensis*)

Some of the most exciting advances in biology are how the microbiome affects host functions and behaviors. In all biological systems, the host and their microbiome make up the holobiont, this symbiotic relationship has coevolved over time [39]. Whether the host genetic variation shapes the host‐associated microbiome in the vertebrates is unclear. The gut microbiota of most vertebrates is dominated by the phyla *Firmicutes* and *Bacteroidetes*. However, the gut microbiota of fish is dominated by the phyla *Proteobacteria* [40]. Our data showed that the gut microbiota of Chinese softshell turtle (*P. sinensis*) was dominated by *Proteobacteria, Bacteroidetes*, and *Firmicutes* (Figure 1D and Table S2). And the gut microbiome of sea turtles (Testudines, Reptilia) and Hawaiian green turtles (*Chelonia mydas*) was dominated by *Proteobacteria, Bacteroidetes*, and *Firmicutes* [41, 42]. While the gut microbiome of juvenile green turtles (*C. mydas*) was dominated by *Bacteroidetes* and *Firmicutes* [43]. *P. sinensis* located between ectothermic amniotic animals (fishes and amphibians) and endothermic amniotic animals (birds and mammals) play an important role in the evolution of vertebrates [18, 19]. Those results indicated a link between host evolutionary relationships and gut microbial structures.

The animal skin is covered by an abundant and diversified microbiome. Most skin microbiome studies focused on humans, domestic animals, and amphibians. Few studies had explored the skin microbiome of aquatic animals, birds, and fish. Nonhuman mammals, including cattle, raccoons, squirrels, pigs, dogs, and sheep, were dominated by *Micrococcus* and *Staphylococcus* [44]. The skin microbiome of birds is influenced by geographic location, sex, diet, captivity. For example, the feathers from caged zebra finches harbored *Bacillus*, which was classified as “feather‐degrading bacteria” [45]. The skin microbiota of scavenger New World vultures was dominated by *Clostridia* and *Fusobacteria* [46]. The amphibian skin microbiome was dominated by *Acidobacteria, Actinobacteria, Bacteroidetes, Cyanobacteria, Firmicutes*, and *Proteobacteria* [47]. The fish skin microbiome was dominated by *Proteobacteria, Firmicutes*, and *Actinobacteria* [48]. The skin microbiome of the Chinese softshell turtle (*P. sinensis*) was dominated by the phylum of *Proteobacteria, Bacteroidetes*, and *Actinobacteria* and genus of *Acinetobacter, Aeromonas*, and *Rothia* (Figure 1D and Table S3). It is necessary to explore the skin microbiome from a wide range of animals to understand how an animal coevolves with its microbiome.

### Enrichment of polysaccharides fermenting microbiome and probiotics in the gut under the RFC

Many gut microbes possess multiple enzymes to help host hydrolyzed plant polysaccharides. We observed that the relative abundances of the phylum *Firmicutes* and *Bacteroidetes*, the family *Lachnospiraceae*, the genus *Ruminococcus, Clostridium, Roseburia, Bacteroides, Prevotella, Bifidobacterium, Eubacterium, Faecalibacterium*, and *Lactobacillus* were much enriched in the gut microbiome of the RFC than that of the IPC (Figures 3A and S4). All of these species were related to polysaccharide fermenting, suggesting a more proportion of polysaccharide fermenting gut microbiota by *P. sinensis* in the RFC than that of the IPC. The phylum *Firmicutes* are dominated in the fecal and gut microbiota of herbivorous vertebrates and play an important role in the fermentation of polysaccharides [43, 49]. The cellulolytic bacteria, such as *Ruminococcus* and *Roseburia*, were able to digest polysaccharides to access cellulose fibrils from dietary fiber, which became available to other members through cross‐feeding. Other members can use the soluble solubilized oligosaccharides and polysaccharides, such as butyrate‐producing *Butyrivibrio, Roseburia*, and *Clostridium*, succinate‐producing *Bacteroides* and *Prevotella*, acetate‐producing *Clostridium* [50, 51]. Additionally, the family *Lachnospiraceae*, the genus of *Eubacterium, Faecalibacterium, Lactobacillus* belonging to *Firmicutes*, and the genus of *Bifidobacterium* belonging to *Actinobacteria* can degrade polysaccharides into short‐chain volatile fatty acids (SCFAs) and characteristic of the gut and feces of herbivorous vertebrates [52, 53]. There are many reports about the relationships between SCFA and diet, health, obesity, and metabolic disease, especially in humans and model animals [54, 55]. The influence of diet on *P. sinensis* fitness needs deeper study. However, it was clear that the weight and body size of *P. sinensis* in the RFC was significantly smaller than those of the IPC (Figure 7A and Table S1). There is a general agreement that a host consuming a fat‐rich diet may enhance the relative abundance of *Proteobacteria* and *Clostridia* [56]. The relative abundance of phylum *Proteobacteria* was much enriched in the gut microbiome of the IPC than that of the RFC (Figure 3A). In a word, our results indicated that the *P. sinensis* gut in the RFC consisted of more proportion of microbiome with the capacity to ferment plant cell–wall polysaccharides to produce SCFAs, which promoted energy metabolism and health. In contrast, *P. sinensis* gut in the IPC consisted more proportion of microbiome with the capacity to ferment fat. Consequently, we observed that there were different concentrations of fatty acids and conjugates, monosaccharides in the gut between the RFC and IPC (Figure 6 and Table S10). Moreover, we observed that the hepatic LDs were significantly accumulated in the *P. sinensis* in the RFC (Figure 7B,C). These results indicated that the lipid metabolism and energy homeostasis of *P. sinensis* was significantly different between the two aquaculture systems.

Notably, *Lactobacillus* and *Bifidobacteria* are the most widely used two probiotics for improving host health, which is related to the metabolic production of SCFAs, organic acids, vitamins, bile salts, polyphenol, bacteriocins, and glycerol, and contribute varied biological functions [53]. We observed that the relative abundances of *Bifidobacterium* and *Lactobacillus* were significantly enriched in the gut microbiome of the RFC than that of the IPC (Figure S4B). Overall, it was suggested that the RFC may benefit host fitness by recruiting more proportion probiotics.

### Metabolic collaboration—intimate association between host and microbiome

The interplay study of microbiome and metabolome was believed as the most promising method to evaluate host–microbiome interactions [57]. Here, the gut paired microbiome–metabolome of *P. sinensis* between the IPC and RFC was analyzed. It was found that the top‐altered pathways were supposed to be involved in the alanine, aspartate, and glutamate metabolism, GABAergic synapse, biosynthesis of unsaturated fatty acids, glucagon signaling pathway, TCA cycle, and arginine and proline metabolism (Figure 6C and Table S11). Sufficient feed was usually fed in the IPC, *P. sinensis* could easily obtain enough food. However, limited food was fed in the RFC, *P. sinensis* needs to capture more food from the surrounding environment, which may cause a completely different diet and activity patterns of *P. sinensis* between the IPC and RFC. The gut harbored trillions of microbes, the metabolic activity of the gut microbiome is crucial in maintaining host homeostasis and health through the production of metabolites. Metabolites were the functional output of the host and microbiome interactions [50]. Thus, it is no surprise to observe that two different aquaculture models had a great impact on the microbiome–host metabolism and host fitness, the impact possibly involved in energy homeostasis, lipid metabolism, endocrine system, anti‐inflammatory activity, and so on (Table S11) [58–62]. In fact, it was observed that the contents of lipid droplets (LDs) were significantly increased in the liver of *P. sinensis* in the RFC (Figure 7B,C), which indicated that different aquaculture systems affect the lipid metabolism and energy homeostasis of *P. sinensis*. Consequently, we observed that there was a huge difference in turtle body shape (Figure 7A). Further studies are needed to confirm the microbiome–host interaction.

Specifically, it was observed that *α*‐ketoglutarate was enriched in the gut of *P. sinensis* in the RFC. *α*‐Ketoglutarate is an important intermediate of the tricarboxylate cycle (TCA cycle) and amino acid metabolic pathway [63], which was a “magic” metabolite involved in delaying aging [64], protection against oxidative stress [65], early germ cell development [66], promotes beige adipogenesis, and prevents obesity in middle‐aged mice [60]. Polyunsaturated fatty acids, such as linoleic acid (LA), eicosapentaenoic acid (EPA), and docosahexaenoic acid (DHA), were enriched in the gut of *P. sinensis* in the RFC, which were associated with significant health benefits in vasodilation, antiplatelet aggregation, anti‐inflammation, rheumatoid, and cardiovascular disease [67]. 4‐Aminobutyric acid (GABA) was enriched in the gut of *P. sinensis* in the RFC, which was the most important inhibitory neurotransmitter, dysfunctions in GABA metabolism involved in anxiety and depression [68]. *N*‐acetyl‐d‐mannosamine (NAM) was enriched in the gut of *P. sinensis* in the RFC, which was identified as a potential oral prebiotic metabolite [69]. It was suggested that the RFC might benefit host fitness by improvements in gut metabolism homeostasis.

A major challenge in gut microbial ecology is identifying the microbe members affecting host metabolic processes and response to environmental change. Correlation‐based analysis of paired microbiome–metabolome was commonly conducted to identify the key microbiome responsible for metabolic differences. However, we used MIMOSAs to quantify each microbiota's contribution to metabolite variation [70, 71]. Here, five metabolites, 2‐oxoglutarate/*α*‐ketoglutarate, *N*‐acetyl‐d‐mannosamine, *cis*‐4‐hydroxy‐d‐proline, nicotinamide, and l‐alanine were identified, which were well‐predicted primarily by several species, respectively (Table S13 and Figure 6D,E). Notably, these species were not all the most variable and the most abundant microbiota, although the family of *Streptomycetaceae, Rhodocyclaceae*, and *Peptostreptococcaceae*, the genus of *Nocardioides* and *Cetobacterium* were the potential key contributors (Table S13). Moreover, each well‐predicted metabolite had multiple key species responsible for the metabolic differences. For example, ASVs in the order Myxococcales, family Streptomycetaceae and *Rhodocyclaceae*, genus *Nocardioides* contributed to the increased degradation of *cis*‐4‐hydroxy‐d‐proline, ASVs in class *Betaproteobacteria*, family *Peptostreptococcaceae, Ruminococcaceae, Aeromonadaceae*, and *Lachnospiraceae*, and genus *Bacteroides* and *Pseudomonas* contributed the increased synthesis from 2‐oxoglutarate (Figure 6E and Table S13,14). And some key species were the major contributor to more than one metabolite. For example, *Peptostreptococcaceae* was the key contributor to *N*‐acetyl‐d‐mannosamine and nicotinamide. Altogether, key contributor analysis indicated that the microbiota contributing most significantly to potential shifts in culture‐associated metabolic phenotypes may not be the most variable or abundant microbiota. Each metabolic shift was the product of a multi‐microbiome.

## CONCLUSIONS

These results suggested that *P. sinensis* skin, oral, and gut microbiome occurred significantly structure and function variation between RFC and IPC, and that different body regions respond differently. Moreover, different aquaculture systems have a significant effect on the host metabolism and fitness of *P. sinensis*. How the effect is transmitted to human nutritional intake through the food chain remains to be further explored. Our results provide new insight into the influence of aquaculture models on the host‐associated microbiome assembly, diversity, and stability, and highlight the key microbiome and gene contributions to metabolite variation in the gut microbiome–metabolome association. Unraveling the host‐associated microbiome in various aquaculture systems may enhance the management of the aquaculture practice toward more sustainable blue foods.

## METHODS

### Study design and processing

The geographic location of the experiment is Yingtan city of China (28.3752°N, 116.9917°E). In May, 6‐month‐old *P. sinensis* were transferred from the GC to the pond and paddy fields for the IPC and RFC, respectively (Figure 1A and Table S1). Ninety Chinese softshell turtles were sampled, *P. sinensis*. In detail, *P. sinensis* were hatched and cultured in a greenhouse for 6 months. In May 2019, 6‐month‐old *P. sinensis* were transferred into the pond and paddy field for intensive pond and RFC model, respectively. Ten 6‐month‐old *P. sinensis* were collected in May 2019 and eighty 12‐month‐old *P. sinensis* were collected from the pond and paddy fields in November 2019. The entire process of sampling, transportation, and DNA extraction was strictly aseptic to avoid environmental microbial contamination. General features of *P. sinensis* in two aquaculture systems were measured (Table S1). The location of the duodenum, ileum, colon, and rectum was identified using hematoxylin–eosin (H&E)‐stained tissue sections (Figure S1).

In the greenhouse, pond, and paddy field groups, 10 animals per group were used to extract the skin (back, limb, and abdomen), oral, and gut (duodenum, ileum, colon, rectum, and whole gut) microbiome DNA (*n* = 240). *P. sinensis* oral and skin (back, limb, and abdomen) microbiome was swabbed to sample the oral and skin microbial communities using sterile Catch‐All Sample Collection Swabs (Epicentre Biotechnologies). Swabs were firmly pressed against the skin or oral area, rubbed back and forth 20 times, then immediately placed into MoBio Power Bead tubes (MoBio Laboratories) and stored at −80°C before oral and skin DNA extraction. *P. sinensis* was dissected under sterile conditions. The segments of the duodenum, ileum, colon, and rectum segments were cut using sterile scissors, and the duodenum, ileum, colon, and rectum contents were obtained. Each sample was stored at −80°C for DNA and metabolite extraction.

For the whole gut contents in the pond and paddy field groups, three whole gut content samples were pooled into one sample, giving twenty whole gut content samples. Ten samples per group were used to extract the whole gut microbiome DNA and metabolites, respectively. Each sample was stored at −80°C for DNA and metabolite extraction.

### DNA extraction and 16S rRNA gene sequencing

DNA was extracted from the swab and gut content samples (*n* = 260 samples) using the DNeasy PowerSoil DNA Isolation kit (QIAGEN) following the manufacturer's protocol. DNA was quantified with a Qubit Fluorometer by using a Qubit dsDNA BR assay kit (Invitrogen), and the quality was checked by running an aliquot on a 1% agarose gel.

A 16S rRNA gene amplification was performed for archaea and bacteria. Barcoded primers targeting the variable V4 regions of the 16S rRNA genes were used for amplification using the universal primer pairs, 515F (GGACTACNVGGGTWTCTAAT) and 806R (GGACTACHVGGGTWTCTAAT) [72–75]. Forward and reverse primers were tagged with Illumina adapter, pad, and linker sequences. Polymerase chain reaction (PCR) enrichment was performed in a 50 μl reaction containing a 30‐ng template, 2 μl fusion PCR primer (10 μM final concentration), and the PCR master mix. The PCR cycling conditions were as follows: 95°C for 3 min, 30 cycles of 95°C for 45 s, 56°C for 45 s, 72°C for 45 s, and final extension for 10 min at 72°C for 10 min. Blank extraction without a sample (negative controls) was also processed in PCR amplification. The PCR products were purified using Agencourt AMPure XP beads and eluted in the elution buffer. The libraries were qualified by the Agilent Technologies 2100 bioanalyzer. The validated libraries were used for sequencing on the Illumina HiSeq. The 2500 platform following the standard Illumina pipelines, and 2 × 250 bp paired‐end reads were generated (Novogene Bioinformatics Institute).

### Microbiome data processing

Amplicon sequences were analyzed using the QIIME2 version 2019.7 [76] and EasyAmplicon [77]. The QIIME2‐DADA2 plugin was employed to denoise the sequences. Sequences were classified taxonomically using the Greengenes 13.8 database [78]. Statistical analyses of the 16S rRNA microbiome sequencing data, such as Kruskal–Wallis, UniFrac, and PERMANOVA, were conducted in the QIIME2 environment, R version 3.5.1 (https://cran.r‐project.org/). The co‐occurrence networks were inferred based on the Spearman correlation matrix constructed with R using the “igraph” package and layout set by ggClusterNet (https://github.com/taowenmicro/ggClusterNet/). Microbial community ecology was analyzed using the “microeco” package [79]. To meet assumptions of homogeneity of variance, data were log‐transformed when required. LEfSe was used to identify differences in taxa composition [80]. The *α* value of 0.05 was used with a threshold on the logarithmic score of LDA being ≥2.0. Tax4Fun2 was used to predict functional redundancies based on 16S rRNA data [81]. Part of the data was visualized by using ImageGP [82].

To estimate the source of the microbial community, Bayesian microbial source tracking was performed using Source Tracker [83]. Sourcetracker was originally developed to infer the proportion of sequences in a “sink” community originating from multiple “source” communities. We combined data for source and sink data sets, the source data was gut, skin, and oral microbiome in the greenhouse, the sink data was the skin (back, limb, and abdomen), oral, and gut (duodenum, ileum, colon, and rectum) microbiome from the pond and rice–fish culture system. It uses Gibbs sampling to assign each sequence in a sink community to a likely source community based on its abundance in each source. All samples were rarefied to 1000 sequences, using the default setting of *α* = 0.001. If <1000 sequences were available for a sample, then all sequences in the sample were used.

The community assembly mechanism was calculated using phylogenetic bin‐based null model analysis (iCAMP) [84]. Briefly, iCAMP includes several key steps: phylogenetic binning; bin‐based null model simulations with phylogenetic diversity for partitioning selection, and taxonomic diversity for partitioning dispersal and drift; then, statistical analysis for assessing the relative importance of different ecological processes (homogeneous selection, heterogeneous selection, dispersal limitation, homogenizing dispersal, drift [and others]) and linking the processes with different environmental factors; finally, the relative abundance of bins governed by each process was aggregated to evaluate its influence on entire community assembly.

The generalists and specialists of the microbiome communities were identified based on the Levins' niche breadth index (*B*), $B_{j}=1/\sum_{i=1}^{N}P_{ij}^{2}$ [32, 35]. The *B_j_* value represents the niche breadth of ASV_*j*_ in a community. *N* is the total number of species across the community, and *P_ij_* is the relative abundance of ASV_*j*_ in community *i* [31]. For a given taxon, it was categorized into specialist or generalist corresponding to *B* > 3 or *B* < 1.5. The index of habitat niche breadth (Bcom) is calculated as the average of *B* values from all taxa occurring in one community.

### Histology

Gut and liver segments were fixed in 4% paraformaldehyde at room temperature for 24 h, followed by an additional 24 h at 4°C, washed four times with phosphate buffered saline, and embedded in paraffin [85, 86]. Five‐mm‐thick sections were cut and stained with H&E and oil red O for gut and liver segment structural analysis using the Leica DMI 6000B fluorescence microscope, respectively.

### Metabolite extractions and liquid chromatography–mass spectrometry (LC‐MS) analysis

To extract metabolites from the samples, 1 ml cold extraction solvent methanol/acetonitrile/H_2_O (2:2:1, v/v/v) was added to the 80 mg sample, and adequately vortexes. After vortexing, the samples were incubated on ice for 20 min, and then centrifuged at 14,000*g* for 20 min at 4°C, the supernatant was collected and flowed through a 96‐well protein precipitation plate. Then, the elution was collected and dried in a vacuum centrifuge at 4°C. For LC‐MS analysis, the samples were redissolved in 100 μl acetonitrile/water (1:1, v/v) solvent and transferred to LC vials.

For the untargeted metabolomics of polar metabolites, extracts were analyzed using a quadrupole time‐of‐flight mass spectrometer (Sciex TripleTOF 6600) coupled to hydrophilic interaction chromatography via electrospray ionization in Shanghai Applied Protein Technology Co. Ltd. The LC separation was on an ACQUIY UPLC BEH Amide column (2.1 mm × 100 mm, 1.7 μm particle size; Waters) using a gradient of solvent A (25 mM ammonium acetate and 25 mM ammonium hydroxide in water) solvent B (acetonitrile). The gradient was 85% B for 1 min and was linearly reduced to 65% in 11 min, and then was reduced to 40% in 0.1 min and kept for 4 min, and then increased to 85% in 0.1 min, with a 5 min re‐equilibration period employed. The flow rate was 0.4 ml/minute, the column temperature was 25°C, the autosampler temperature was 5°C, and the injection volume was 2 μl. The mass spectrometer was operated in both negative ion and positive ionization modes. The electrospray ionization source conditions were set as follows: Ion Source Gas1 (Gas1) as 60, Ion Source Gas2 (Gas2) as 60, curtain gas (CUR) as 30, source temperature: 600°C, IonSpray Voltage Floating (ISVF) ± 5500 V. In MS acquisition, the instrument was set to acquire over the *m*/*z* range of 60–1000 Da, and the accumulation time for time‐of‐flight mass spectrometry (TOFMS) scan was set at 0.20 s/spectra. In auto MS/MS acquisition, the instrument was set to acquire over the *m*/*z* range of 25–1000 Da, and the accumulation time for production scan was set at 0.05 s/spectra. The production scan is acquired using information‐dependent acquisition (IDA) with high sensitivity mode selected. The parameters were set as follows: the collision energy (CE) was fixed at 35 V with ±15 eV; declustering potential (DP), 60 V (+) and −60 V (−); exclude isotopes within 4 Da, candidate ions to monitor per cycle: 10.

### Metabolomics data processing

The raw MS data (wiff. scan files) were converted to MzXML files using ProteoWizard MSConvert before importing into freely available XCMS software. For peak picking, the following parameters were used: centWave *m*/*z* = 25 ppm, peak width = *c* (10, 60), and prefilter = *c* (10, 100). For peak grouping, bw = 5, mzwid = 0.025, and minfrac = 0.5 were used. In the extracted ion features, only the variables having more than 50% of the nonzero measurement values in at least one group were kept. Metabolites were unambiguously identified by comparing the accuracy of *m*/*z* value and MS/MS spectra with an in‐house database established with 3000 authentic standards (Shanghai Applied Protein Technology) and by metabolic reaction network (MRN)‐based recursive algorithm [87]. After normalized to total peak intensity, the processed data were uploaded before importing into SIMCA‐P (version 14.1, Umetrics), where it was subjected to multivariate data analysis, including Pareto‐scaled principal component analysis and orthogonal partial least‐squares discriminant analysis (OPLS‐DA). Significance was determined using an unpaired Student's *t* test. VIP value >1 and *p* < 0.05 were considered statistically significant. The MSEAs, as well as MetPA on all identified metabolites, were performed using an online platform, MetaboAnalystR (https://www.metaboanalyst.ca/MetaboAnalyst/home.xhtml) [20, 21, 88].

### MIMOSA integrative Microbiome–Metabolite data analysis

MIMOSAs were used to identify metabolites whose variation across samples can be explained by variation in the metabolic potential of the microbiome [70, 71]. Each metabolomics data set separately was then analyzed using the R package *MIMOSA* version 1.0.1 (http://elbo.gs.washington.edu/software_MIMOSA.html). PICRUSt output from a 16S rRNA table was used. Then the PICRUSt output is paired with reaction information to estimate the CMP scores for each sample and metabolite, using a local FDR *q* < 0.01. Variation in predicted CMP scores is compared to variation in measured metabolite abundances (using pairwise differences) to identify well‐predicted (consistent) and antipredicted (*contrasting*) metabolites. Well‐predicted metabolites are defined as those for which variation in CMP scores is significantly correlated (using a Mantel test) with variation in measured metabolite abundance at an FDR of 0.01. Antipredicted metabolites are similarly defined as those for which the variation in CMP scores is significantly negatively correlated with variation in measured metabolite abundances (FDR 0.01). Metabolic categorization is based on KEGG data. A perturbation‐based approach is used to additionally identify key species, genes, and reaction contributors to CMP scores [71].

## AUTHOR CONTRIBUTIONS

Xia Ding conceived and designed the study, carried out the data analysis, and drafted the manuscript. Feng Jin contributed to sampling and data acquisition. Jiawang Xu, Shulei Zhang, Dongxu Chen, and Beijuan Hu contributed to the data acquisition and carried out the data analysis. Yijiang Hong conceived and designed the study. All authors read and approved the final manuscript.

## CONFLICTS OF INTEREST

The authors declare no conflicts of interest.

## Supporting information

Supplementary information.

Supplementary information.

Supplementary information.

Supplementary information.

## Data Availability

Raw data and sample metadata are publicly available under the NCBI SRA database with the accession number as No. PRJNA766351.
