# Correction to “The impact of aquaculture system on the microbiome and gut metabolome of juvenile Chinese softshell turtle (*Pelodiscus sinensis*)”

**DOI:** 10.1002/imt2.151

**Published:** 2023-11-06

In Ding et al. [1], the following ethics statement should have been added.

The ethics application (No. NCULAE‐20181121637) was approved by the Institutional of Animal Care and Use Committees of Nanchang University.

The authors wish to apologize for the oversight.
