# Supplementary material for: The impact of aquaculture system on the microbiome and gut metabolome of juvenile Chinese softshell turtle (Pelodiscus sinensis)
Source: Imeta. 2022 Apr 5;1(2):e17. doi: 10.1002/imt2.17 (PMC10989827; doi:10.1002/imt2.17)
Supplement: Supplementary file 4 — Supplementary information. [file IMT2-1-e17-s001.docx]

**SUPPORTING INFORMATION**

**Supplementary Figures in one word file.**

**Figure S1** Histology of the duodenum, ileum, colon, rectum of *Pelodiscus sinensis*. E-stained sections are shown. Scale bar: 500 µm, 200 µm, 50 µm.

**Figure S2** Venn diagram of shared and unique ASVs among the body regions.

**Figure S3** Hub (keystone) microbiome species within the co-occurrence network. Networks are colored by phylum and sized based on the hub score of the ASV.

**Figure S4** Linear discriminant analysis (LDA) effect size (LEfSe) analysis revealed significant microbial differences in the gut between the pond and rice-fish culture model. (A) LDA scores > 3.5 and *P <* 0.05 are shown. (B) LEfSe analysis at the genus level.

**Figure S5** Metabolite concentrations across samples

**Figure S6** Metabolite Predictions by Category

**Figure S7** The physical parameters of water in the RFC and IPC. Data were log10 transformed.

**Supplementary Tables in one excel file.**

**Table S1** The holotype of *Pelodiscus* *sinensis* in two aquaculture systems.

**Table S2** Relative abundance of the microbiome at the phylum level.

**Table S3** Relative abundance of the microbiome at the genus level.

**Table S4** Relative abundances of the top eighty genus species in each body region from different cultural models and ages.

**Table S5** Permutational MANOVA results using Bray-Curtis as a distance metric.

**Table S6** Pairwise permanova results

**Table S7** Network property of co-occurrence networks

**Table S8** Estimated source of microbial communities

**Table S9** Quantification of the relative roles of deterministic processes (homogeneous and heterogeneous selection), stochastic processes (dispersal limitation, homogenizing dispersal and drift)

**Table S10** Main-class of KEGG pathway enrichment analysis

**Table S11** Metabolic pathways undergoing significant changes between the pond and rice-fish culture systems

**Table S12** Gene/Reaction Contributors Analysis

**Table S13** Metabolites consistent with metabolic potential

**Table S14** Contributing ASVs for each meta

**Table S15** The physical parameters of water in the RFC and IPC
