# Supplementary material for: The impact of aquaculture system on the microbiome and gut metabolome of juvenile Chinese softshell turtle (Pelodiscus sinensis)
Source: Imeta. 2022 Apr 5;1(2):e17. doi: 10.1002/imt2.17 (PMC10989827; doi:10.1002/imt2.17)
Supplement: Supplementary file 3 [file IMT2-1-e17-s002.docx]

Figure S1

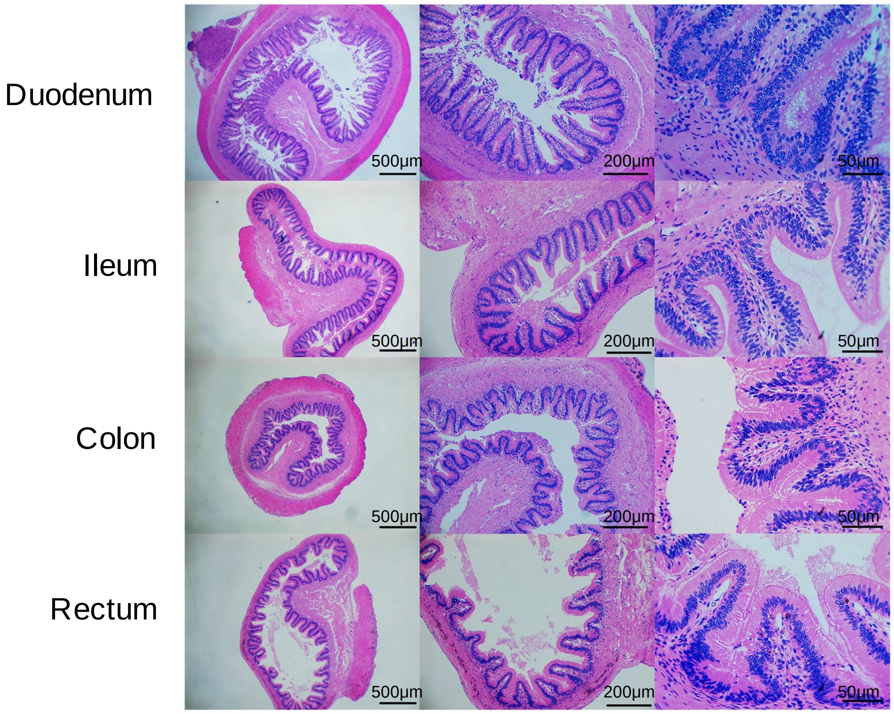

Figure S2

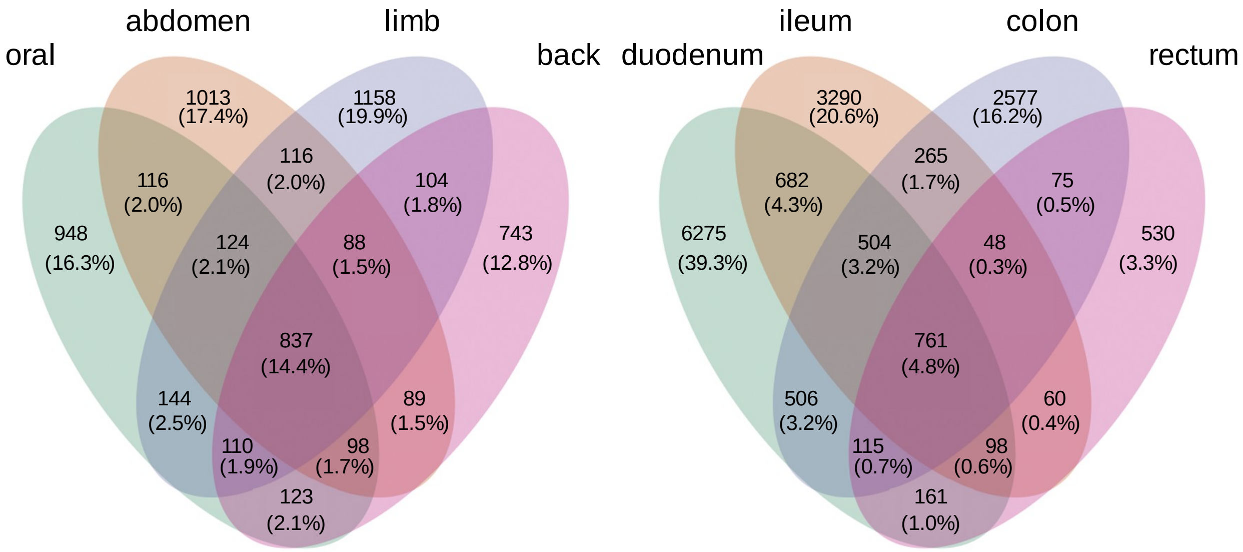
oral
abdomen
limb
back
1013
(17.4%)
1158
(19.9%)
116
(2.0%)
116
(2.0%)
104
(1.8%)
948
(16.3%)
124
(2.1%)
88
(1.5%)
743
(12.8%)
837
(14.4%)
144
(2.5%)
89
(1.5%)
110
(1.9%)
98
(1.7%)
123
(2.1%)
duodenum
ileum
colon
rectum
3290
(20.6%)
2577
(16.2%)
265
(1.7%)
682
(4.3%)
75
(0.5%)
6275
(39.3%)
504
(3.2%)
48
(0.3%)
530
(3.3%)
761
(4.8%)
506
(3.2%)
60
(0.4%)
115
(0.7%)
98
(0.6%)
161
(1.0%)

Figure S3

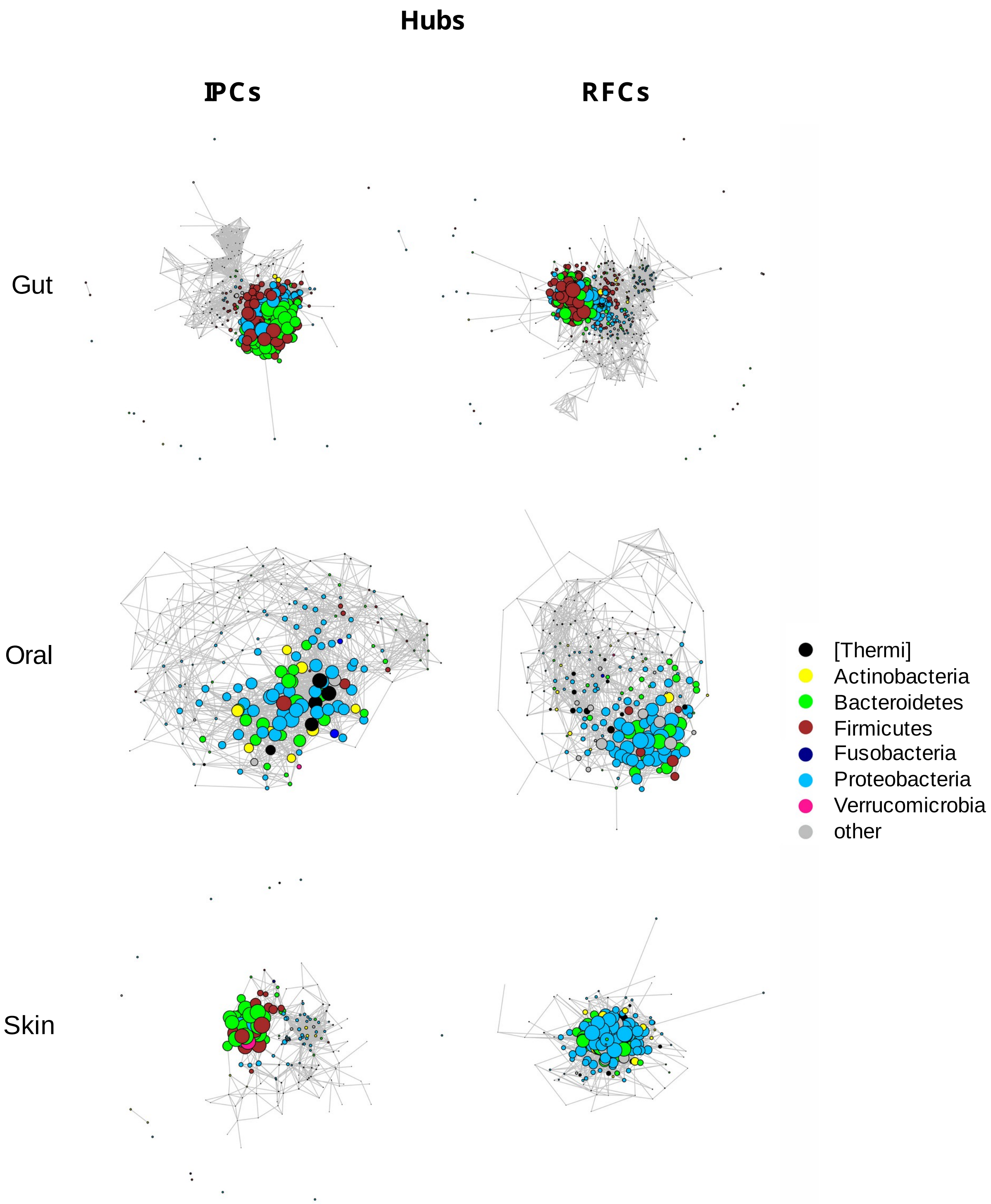
Hubs
IPCs
RFCs
Gut
Oral
Skin
[Thermi]
Actinobacteria
Bacteroidetes
Firmicutes
Fusobacteria
Proteobacteria
Verrucomicrobia
other

Figure S4

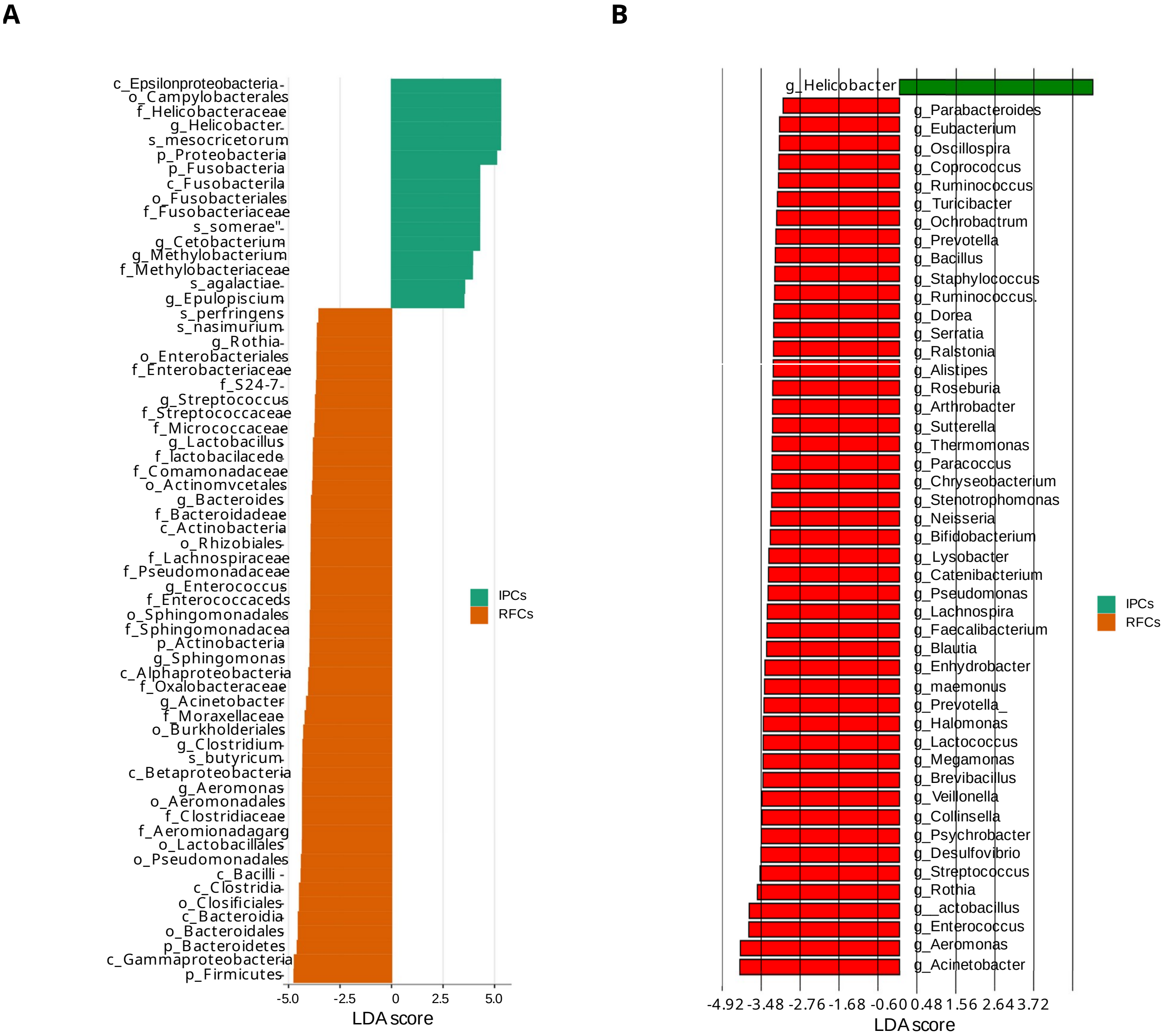

A
B
c_Epsilonproteobacteria
o_Campylobacterales
f_Helicobacteraceae
g_Helicobacter
s_mesocricetorum
p_Proteobacteria
p_Fusobacteria
c_Fusobacteria
o_Fusobacteriales
f_Fusobacteriaceae
s_somerae"
g_Cetobacterium
g_Methylobacterium
f_Methylobacteriaceae
s_agalactiae
g_Epulopiscium
s_perfringens
s_nasimurium
g_Rothia
o_Enterobacteriales
f_Enterobacteriaceae
f_S24-7
g_Streptococcus
f_Streptococcaceae
f_Micrococcaceae
g_Lactobacillus
f_lactobacilacede
f_Comamonadaceae
o_Actinomvcetales
g_Bacteroides
f_Bacteroidadeae
c_Actinobacteria
o_Rhizobiales
f_Lachnospiraceae
f_Pseudomonadaceae
g_Enterococcus
f_Enterococcaceds
o_Sphingomonadales
f_Sphingomonadacea
p_Actinobacteria
g_Sphingomonas
c_Alphaproteobacteria
f_Oxalobacteraceae
g_Acinetobacter
f_Moraxellaceae
o_Burkholderiales
g_Clostridium
s_butyricum
c_Betaproteobacteria
g_Aeromonas
o_Aeromonadales
f_Clostridiaceae
f_Aeromionadagarg
o_Lactobacillales
o_Pseudomonadales
c_Bacilli
c_Clostridia
o_Closificiales
c_Bacteroidia
o_Bacteroidales
p_Bacteroidetes
c_Gammaproteobacteria
p_Firmicutes
-5.0
-2.5
0
2.5
5.0
LDA score
IPCs
RFCs
g_Helicobacter
g_Parabacteroides
g_Eubacterium
g_Oscillospira
g_Coprococcus
g_Ruminococcus
g_Turicibacter
g_Ochrobactrum
g_Prevotella
g_Bacillus
g_Staphylococcus
g_Ruminococcus.
g_Dorea
g_Serratia
g_Ralstonia
g_Alistipes
g_Roseburia
g_Arthrobacter
g_Sutterella
g_Thermomonas
g_Paracoccus
g_Chryseobacterium
g_Stenotrophomonas
g_Neisseria
g_Bifidobacterium
g_Lysobacter
g_Catenibacterium
g_Pseudomonas
g_Lachnospira
g_Faecalibacterium
g_Blautia
g_Enhydrobacter
g_maemonus
g_Prevotella_
g_Halomonas
g_Lactococcus
g_Megamonas
g_Brevibacillus
g_Veillonella
g_Collinsella
g_Psychrobacter
g_Desulfovibrio
g_Streptococcus
g_Rothia
g__actobacillus
g_Enterococcus
g_Aeromonas
g_Acinetobacter
-4.92
-3.48
-2.76
-1.68
-0.60
0.48
1.56
2.64
3.72
LDA score
IPCs
RFCs

Figure S5

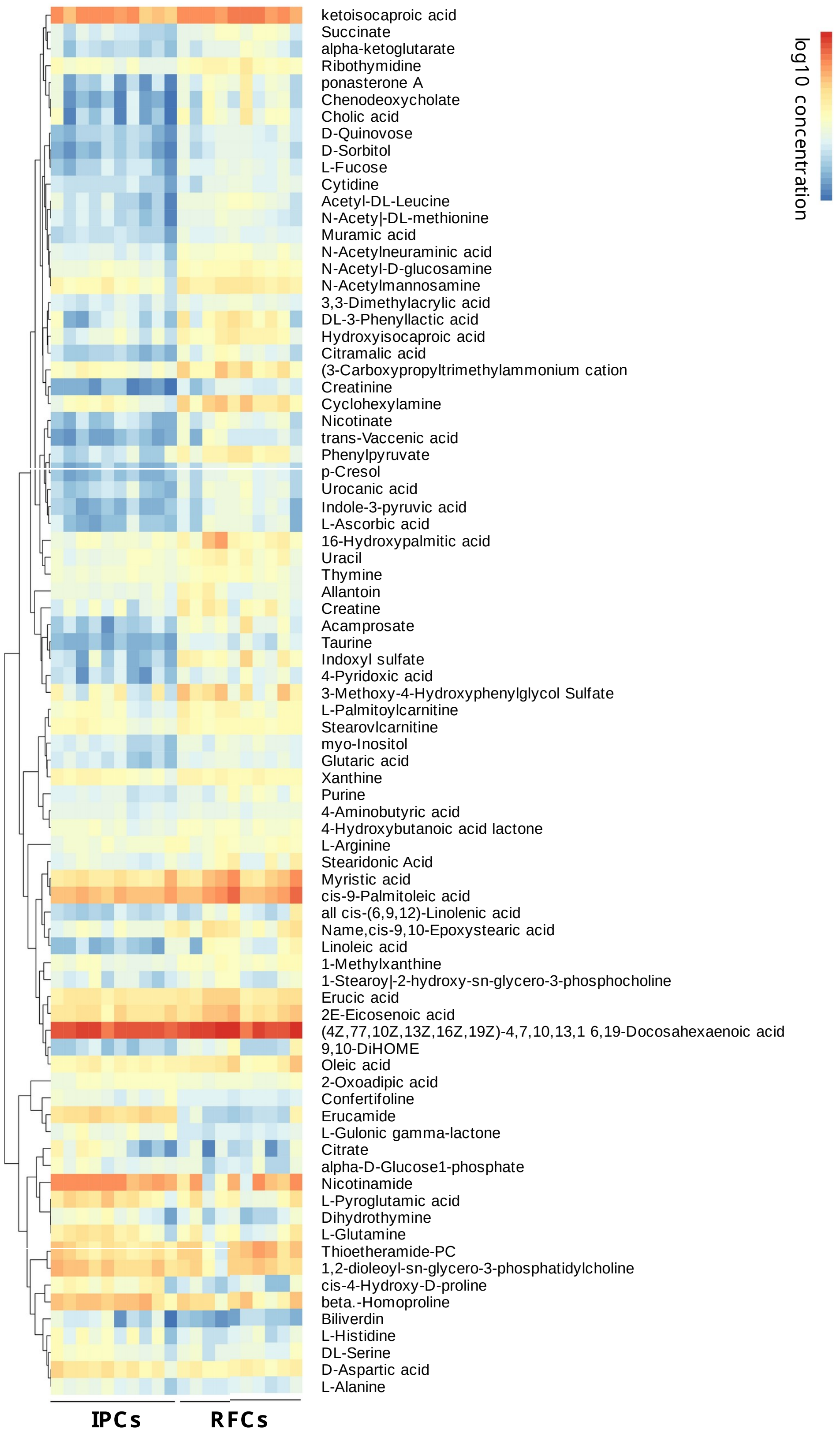

ketoisocaproic acid
Succinate
alpha-ketoglutarate
Ribothymidine
ponasterone A
Chenodeoxycholate
Cholic acid
D-Quinovose
D-Sorbitol
L-Fucose
Cytidine
Acetyl-DL-Leucine
N-Acety|-DL-methionine
Muramic acid
N-Acetylneuraminic acid
N-Acetyl-D-glucosamine
N-Acetylmannosamine
3,3-Dimethylacrylic acid
DL-3-Phenyllactic acid
Hydroxyisocaproic acid
Citramalic acid
(3-Carboxypropyltrimethylammonium cation
Creatinine
Cyclohexylamine
Nicotinate
trans-Vaccenic acid
Phenylpyruvate
p-Cresol
Urocanic acid
Indole-3-pyruvic acid
L-Ascorbic acid
16-Hydroxypalmitic acid
Uracil
Thymine
Allantoin
Creatine
Acamprosate
Taurine
Indoxyl sulfate
4-Pyridoxic acid
3-Methoxy-4-Hydroxyphenylglycol Sulfate
L-Palmitoylcarnitine
Stearovlcarnitine
myo-Inositol
Glutaric acid
Xanthine
Purine
4-Aminobutyric acid
4-Hydroxybutanoic acid lactone
L-Arginine
Stearidonic Acid
Myristic acid
cis-9-Palmitoleic acid
all cis-(6,9,12)-Linolenic acid
Name,cis-9,10-Epoxystearic acid
Linoleic acid
1-Methylxanthine
1-Stearoy|-2-hydroxy-sn-glycero-3-phosphocholine
Erucic acid
2E-Eicosenoic acid
(4Z,77,10Z,13Z,16Z,19Z)-4,7,10,13,1 6,19-Docosahexaenoic acid
9,10-DiHOME
Oleic acid
2-Oxoadipic acid
Confertifoline
Erucamide
L-Gulonic gamma-lactone
Citrate
alpha-D-Glucose1-phosphate
Nicotinamide
L-Pyroglutamic acid
Dihydrothymine
L-Glutamine
Thioetheramide-PC
1,2-dioleoyl-sn-glycero-3-phosphatidylcholine
cis-4-Hydroxy-D-proline
beta.-Homoproline
Biliverdin
L-Histidine
DL-Serine
D-Aspartic acid
L-Alanine
IPCs
RFCs
log10 concentration
7
6
5
4

Figure S6

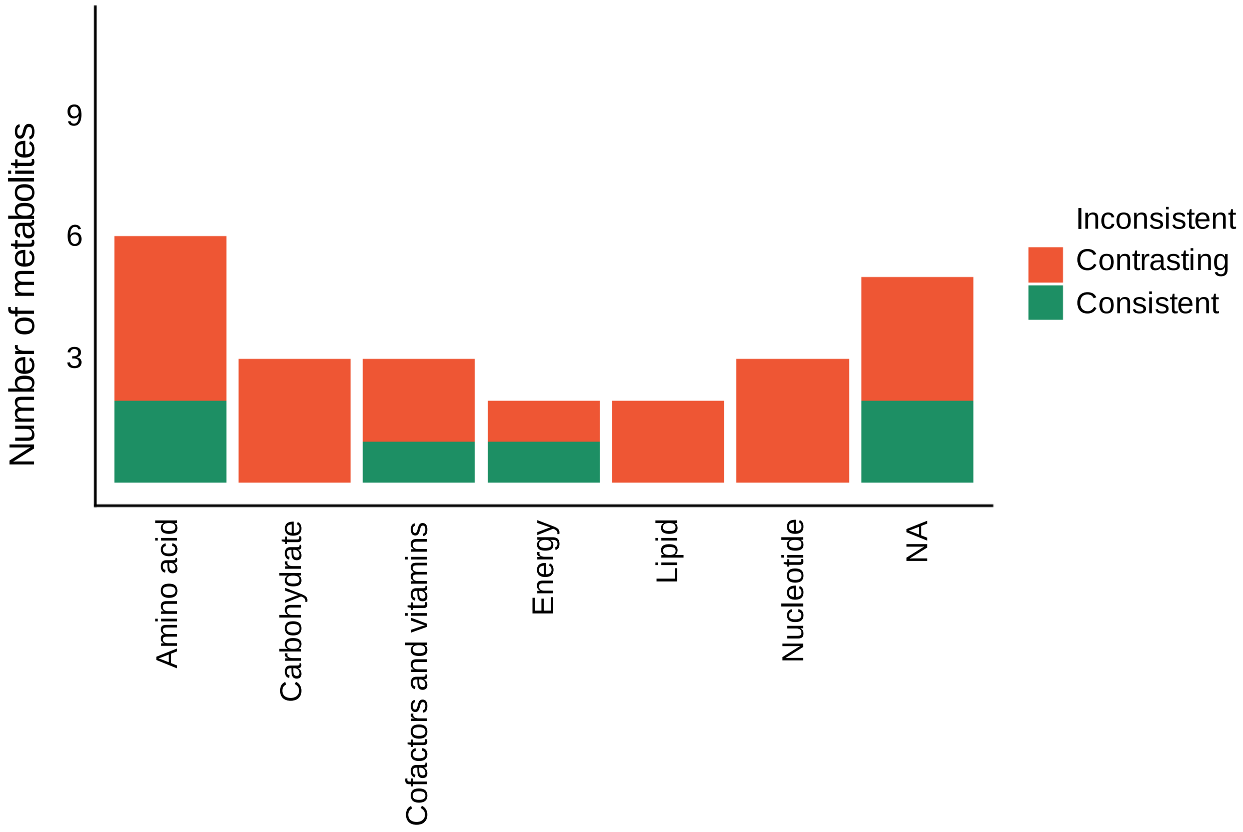

Number of metabolites
9
6
3
Amino acid
Carbohydrate
Cofactors and vitamins
Energy
Lipid
Nucleotide
NA
Inconsistent
Contrasting
Consistent

Figure S7

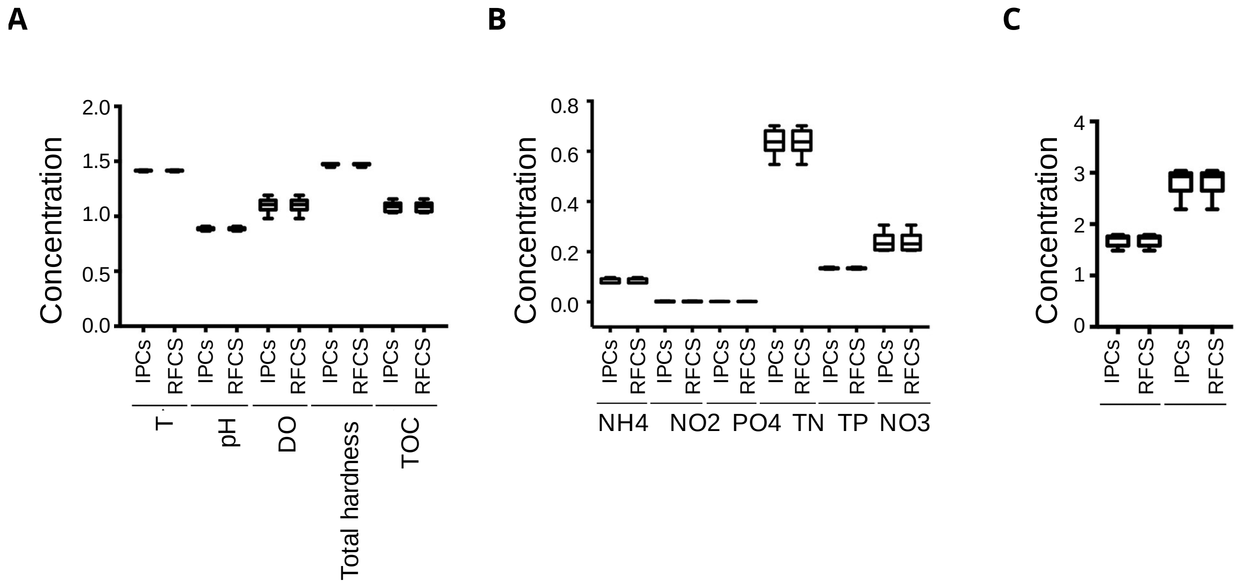
A
Concentration
2.0
1.5
1.0
0.5
0.0
IPCs
RFCS
T
pH
DO
Total hardness
TOC
B
Concentration
0.8
0.6
0.4
0.2
0.0
NH4
NO2
PO4
TN
TP
NO3
C
Concentration
4
3
2
1
0
